# The Treatment of Ringworm of the Scalp

**Published:** 1906-08-11

**Authors:** 


### The General Practitioner's Column.

[Contributions to this Column are invited, and if accepted will be paid for.]

### THE TREATMENT OF RINGWORM OF THE SCALP.

By Harry G. Critchley, M.A., M.D.

At the present time the occurrence of ringworm has become so extensive and persistent that it has earned for itself a large degree of importance in medical and social economics.

I have read and re-read the article by Mr. J. S. Mellish in your issue of July 30, and, as an enormous amount of ringworm comes under my notice throughout the year, I should hail with delight and welcome as an advance any medicinal agent which would be efficacious and painless. The statement that he has successfully treated over 200 cases with a single application of formalin is likely to arouse much interest, for I cannot but feel that the experience of Mr. Mellish is remarkable, especially in view of the fact that he writes respecting tinea tonsurans and not the circinata. The treatment by formalin is, however, not new. My experience of formalin is that it frequently fails, even after prolonged treatment, and that it, like all medicinal agents, causes more or less painful erythema, or even vesication, for any remedy in order to be efficacious must penetrate four millimetres into the skin, and this cannot readily be done, except by the agency of *x*-rays, without destroying, temporarily at least, a considerable thickness of the cuticle. The treatment by *x*-rays is certainly the most efficacious, economical, cleanly, and pleasant. I have heard and read of a few cases where this treatment has set up severe dermatitis, and even chronic alopecia, but they were among the earliest ones; and M. Sabouraud, who met with six such cases in his own practice, has done 4,000 consecutive ones without any such untoward event. I do not rely entirely upon the reaction of the Sabouraud pastille, but have also regard to the register of the ampère and milliampère meters, and by these means am able to calculate the " dose " to the finest possible nicety.

Mr. Mellish quotes Sabouraud's remarks that the cost of ringworm treatment has been reduced from 2,000 to 260 francs per case, and compares this with the cost of a bottle of formalin. Such comparison is ridiculously illogical. M. Sabouraud's figures had reference to the average cost of maintenance in the Ecole Lailler of the St. Louis Hospital, and not to the cost of the treatment, which is infinitesimal.

Quite recently I have treated a child whose condition had resisted various forms of treatment for two years without improvement; she had one application of *x*-rays, and on the twenty-first day afterwards received a clean bill of health, while another which had specially declined to be influenced by formalin, having received a single application of *x*-rays, was free from infection in twenty-three days.

A Reply by J. Stafford Mellish, M.R.C.S., L.R.C.P. (Lond.).

In reply to Dr. H. G. Critchley's comments on my article, I would say that, though not new, as far as I can judge from what I have seen on the subject, formalin has been used more as a paint for ringworm and not rubbed in to any extent; on this *rubbing in* I lay great stress, as I agree with Dr. Critchley that it is necessary for any agent to enter the skin for some depth to be efficacious; in fact, any curative agent to cure a disease must get to the seat of that disease, and by rubbing you remove crusts, open pustules and follicles, and admit the formalin and its vapour; on the same principle you poultice to remove scabs in eczema before applying dressings, and on the same line of argument as far as ringworm is concerned, an agent which is of the nature of a spirit is bound to be more efficacious than one of an oily nature, though possibly of the same germicidal strength, because the spirit is of a more searching and permeating nature than an oil which tends to close the pores.

Of course, formalin does produce some erythema and vesication, but is not very painful, and the smarting only lasts a few minutes and does not lessen the cure. Up to now I have not had any severe dermatitis or chronic alopecia, which are admittedly caused occasionally by *x*-rays, either of which, while perhaps not mattering so much in a hospital, would be a very serious mishap in private practice. With regard to my comparison of the price of a bottle of formalin with the cost of Dr. Sabourand's cases (of 2,000 francs formerly and by *x*-rays 260 francs per case), which Dr. Critchley assures us is " ridiculously illogical " because M. Sabourand's figures refer to the maintenance of the case, surely it does not matter whether the money is spent for the medical agent used or for food or other incidental expense. The " case " costs the ratepayer in our unions or the Board of Management at hospitals so much—say £10 in food and support while under treatment. Now if this same case can be treated at home and cured with an application or two of formalin, and is therefore not putting the ratepayer or charitable organisation to any expense, then I say the comparison at any rate is practical, and the balance is in favour of the formalin treatment from the public's point of view, if not from the scientist's, and it would puzzle Dr. Critchley to carry about an *x*-ray outfit in the hilly country districts where one may occasionally " take a toss." I remember well attending nine cases of ringworm in a cottage some eight miles from me. These people live in a place only approachable on foot or horseback, and two stiff stiles to negotiate. Thanks to the efficacy of formalin, two journeys proved sufficient. I well rubbed all the patches the first time, and ten days afterwards I visited them, and touched up two spots that looked suspicious. Three weeks afterwards, on passing, I found they were quite clear. Now, with all seriousness, one could hardly have got an $x$-ray apparatus there, and would have had to kidnap the children to get them near it, and they had no means of getting into a surgery.

I am afraid that $x$-rays is without the scheme of practical medicine in country districts, though, no doubt, very efficacious in a consulting-room. At the same time I am quite satisfied with the results obtained by the use of formalin, and feel that for country work it is well in front of $x$-rays.

### "RUB IT IN."

By Desborough Brodie, M.B., B.S. (Lond.).

There have been various letters and articles in medical papers adversely commenting on the possibility of a cure of ringworm of the scalp with purely local medicinal applications. In every case in which I have used formalin I have found it most satisfactory, but it must always be borne in mind that a mere application or painting is no good. The formalin must be well rubbed in. I cannot make too great a point of this. It is essential. I suppose I must bow to the efficacy of $x$-ray treatment, but I must remind those who are inclined to scoff at local medicinal treatment that they would find it entirely beyond practical medicine in a large, scattered country practice to carry the necessary $x$-ray apparatus hung to the waist, like John Gilpin and his bottles.

And so, again, I strongly urge not only those in a similar position to myself, but also the "workers in towns," to give a fair trial to formalin—*and rub it in.*

